# Ultrastructure of Cells and Microanalysis in *Malus domestica* Borkh. ‘Szampion’ Fruit in Relation to Varied Calcium Foliar Feeding

**DOI:** 10.3390/molecules25204622

**Published:** 2020-10-11

**Authors:** Piotr Kowalik, Tomasz Lipa, Zenia Michałojć, Mirosława Chwil

**Affiliations:** 1 Institute of Horticulture Production, University of Life Sciences in Lublin, Akademicka 15, 20-950 Lublin, Poland; piotrek88.1@wp.pl (P.K.); tomasz.lipa@up.lublin.pl (T.L.); zenia.michalojc@up.lublin.pl (Z.M.); 2 Department of Botany and Plant Physiology, University of Life Sciences in Lublin, Akademicka 15, 20-950 Lublin, Poland

**Keywords:** epidermis, hypodermis, cell wall, cytoplasmic membrane, Ca mapping, apple tree

## Abstract

Calcium is one of the most poorly reutilized nutrients. Its deficiencies cause various physiological disturbances and, consequently, reduce the quantity and quality of yields. Reduced content of Ca^2+^ ions in cells leads to development of, e.g., bitter pit in apples. Efficient and instantaneous mitigation of Ca^2+^ deficiencies is provided by foliar feeding. There are no detailed data on the effect of foliar feeding with various calcium forms on the cell structure or on the microanalysis and mapping of this element in apple fruit cells. Therefore, we carried out comparative studies of the ultrastructure of epidermis and hypodermis cells, to assess the content and distribution of calcium in the cell wall, cytoplasmic membrane, cytoplasm, and precipitates of *Malus domestica* Borkh. ‘Szampion’ fruit exposed to four Ca treatments, including the control with no additional Ca supplementation (I) and foliar applications of Ca(NO_3_)_2_ (II), CaCl_2_ (III), and Ca chelated with EDTA (IV). Light and transmission electron microscopy and an X-ray microanalyzer were used and showed a beneficial effect of calcium preparations on the ultrastructure of fruit epidermis and hypodermis cells, manifested in the presence of a normally developed cell wall with a regular middle lamella, preserved continuity of cytoplasmic membranes, and stabilized cell structure. In the selected elements of apical epidermis cells, the highest level of Ca^2+^ ions was detected in the middle lamella, cell wall, plasmalemma, and cytoplasm. The highest increase in the Ca^2+^ content in these cell constituents was recorded in treatment IV, whereas the lowest value of the parameters was noted in variant III.

## 1. Introduction

Currently, researchers have been increasingly interested in the involvement of calcium ions in plant regulation and development. Calcium is one of the basic plant nutrients. This element not only determines the amount and quality of crops, but also ensures resistance to various types of abiotic and biotic stress factors. Appropriate levels of calcium in soil and plant stabilize many biochemical processes [1,2,3,4].

### 1.1. Role of Calcium in Plants

The calcium content in plants growing on soils with a sufficient level of this element is in the range of 0.1–5% of dry matter. Calcium serves a number of important structural, biochemical, and physiological functions in the plant. It regulates stomatal movements; hence, plants can limit transpiration and use water effectively in unfavorable conditions [5,6,7]. Calcium is a secondary intracellular transmitter of environmental signals. It alleviates the effects of stress, e.g., by neutralization of reactive oxygen species generated in cells [8,9]. Calcium determines directly and indirectly the effective utilization of light, CO_2_, water, and nutrients. Exogenous Ca^2+^ ions play an important role in photosynthesis, as they determine the proper function of photosystem II (PS II-water-plastoquinone oxidoreductase), i.e., the first protein complex located in thylakoid membranes, in the light-dependent reaction of oxygenic photosynthesis, which is responsible for adaptation to stressful conditions [10,11]. Wang et al. [12] showed the protective role of exogenous calcium, suggesting that calcium nitrate enhanced the electron transfer of PSII (especially beyond QA-) and prevented inactivation of reaction centers in salt-stressed tall fescue.

In turn, Tan et al. [13] reported a beneficial effect of application of CaCl_2_ on the photosynthesis process in heat-stressed (43 °C for 2 h) *Nicotiana tabacum* L. plants. This effect was associated with improvement in stomatal conductance and thermostability of the oxygen-evolving complex (OEC), which might be due to lower accumulation of reactive oxygen species. Ca^2+^ pretreatment of heat-stressed tobacco plants decreased the contents of H_2_O_2_ and superoxide radical anion (O_2_^•−^), enhanced the induction of heat shock protein 70 (HSP70), and increased glutathione reductase (GR) activity, whilst the activities of superoxide dismutase (SOD), catalase (CAT), ascorbate peroxidase (APX), and peroxidase (POD) were either enhanced or inhibited, compared to the high-temperature treatment.

The findings reported by Zhang et al. [14] demonstrated that Ca^2+^ participated in the nitric oxide (NO)-induced tolerance to low temperature (11 °C/7 °C) by modulating leaf gas exchange, PSII-related processes, carbohydrate metabolism, and expression of chlorophyll synthesis-related genes in *Cucumis sativus* L. seedlings leaves. Hajihashemi et al. [15] described the protective role of pretreatment of *Chenopodium quinoa* (quinoa) seeds with CaCl_2_, H_2_O_2_, and sodium nitroprusside (SNP) at concentrations of 5, 5, and 0.2 mM, respectively, which limited the adverse effect of salt stress on seed germination. This beneficial effect was manifested by a significant increase in the germination rate, relative germination rate, and germination index, as well as enhanced protein and amino acid contents. Moreover, the stimulated amylase activity resulted in starch breakdown and increased content of water-soluble sugars, which have an osmoprotectant role in overcoming salt stress.

Calcium is a component or an activator of many important enzymes. As part of respiratory enzymes, it determines fruit firmness. Therefore, low calcium content in stored fruit contributes to high intensity of respiratory processes, which leads to rapid turgor loss [8,9]. Ca^2+^ ions regulate the hormonal balance in plants. They reduce the synthesis of ethylene, delay fruit aging, and are involved in carbohydrate metabolism by increasing starch accumulation [6,16,17].

At the cellular level, calcium is a structural component of cell walls linking the long chains of α-D-galacturonic acid in protopectins. Pectins, e.g., calcium pectinate, constitute the main structural fraction of the middle lamella and maintain tissue integrity. The role of pectins is particularly important in the fruit structure. These compounds form a specific “scaffold,” which is solid and strong at high calcium content, but fragile and weak at low Ca^2+^ concentrations. Hence, fruit with low calcium content will be small with poorly developed flesh, low firmness, and a thin epidermis. Calcium dehydrates cytoplasmic colloids, thereby increasing their viscosity and reducing hydrophilicity. It stabilizes cell walls and cytoplasmic membranes and regulates their permeability and selectivity. It also influences cell division, growth, and function, and determines proper pollen germination, pollen tube growth, and seed formation [8,16,18,19].

### 1.2. Foliar Feeding

Besides the basic mechanism of nutrient uptake via the root system, certain amounts of essential elements can also be taken up by shoots, leaves, and fruit, as applied in practice. This type of nutrient supply is used especially in orchard and indoor crop cultivation. Nutrients supplied through foliar feeding penetrate through ectodesmata (approx. 30 nm in diameter) present in the outer cell wall of the shoot epidermis. These routes of penetration of secreted substances to the wall are involved in the formation of cuticle and surface waxes [20,21].

To facilitate the penetration of nutrients through the cuticle, agents increasing adhesion of liquids to plant cells are added to working solutions [22]. Foliar feeding is usually based on chelated fertilizers; however, calcium ions form such compounds with difficulty [20,23,24,25]. A thick cuticle and wax layer on the fruit surface is a barrier that prevents sprayed nutrients from penetration [26,27,28]. A thick apple fruit cuticle built of overlapping wax platelets contributes to lower susceptibility of the epidermis to damage and russeting formed by the cork tissue. In turn, a thin cuticle built of a hardly flexible uniform tissue promotes fruit russeting [29,30,31,32,33,34]. The susceptibility of apples to russet is carried by genes responsible for the structure of the outer fruit layers, in particular the cuticle [35,36].

### 1.3. Calcium Content

Estimation of the calcium content in apples immediately before harvesting is helpful in determination of their suitability for long-term storage. It is estimated that the content of this element should amount to at least 5 mg Ca per 100 g of flesh, i.e., 600–1000 mg kg^−1^ of dry weight [37,38]. The fruit calcium content is determined by both agrotechnical and genetic factors. The rootstock has an impact on the Ca content, which in noble varieties is largely modified by atmospheric conditions prevailing during the vegetation season [39]. ‘Szampion’ apples are susceptible to bitter pit caused mainly by low calcium content. As shown by Neilsen [40], bitter pit develops in apples with a Ca level below 250 mg kg^−1^ d.w. The critical value of this element in ‘Szampion’ apples estimated by Lanauskas and Kviklene [41] is below 380 mg Ca kg^−1^ d.w. Besides the calcium content, the level of potassium, nitrogen, and magnesium (ratio K:Ca, N:Ca, Mg:Ca) provides valuable information about fruit storability. The of K:Ca ratio is the most important relationship between elements. The most favorable value of the ratio in terms of long-term cold storage of apples ranges from 20 to 30, while a wider range of the potassium-to-calcium ratio in apples considerably worsens their suitability for storage, and results in development of bitter pit [42,43]. In turn, the optimal N:Ca ratio in apples is in the range of 10–20, while a higher value of this ratio increases the risk of development of storage-related diseases [43,44].

The accumulation of Ca in the apples is still discussed [17], although a large body of evidence suggests its continuous uptake during the period of fruit growth. Calcium accumulation in apples increases from bloom until ripening [45]; however, as the fruit weight increases at a greater pace, its Ca concentration decreases. It has been evidenced that, due to the very poor reallocation of this element, it is very important to target the fruit when spraying Ca to apple trees, as the amount translocated from the leaves to the fruit is negligible. Foliar Ca feeding results in a far greater increase in the leaf Ca level is than in the fruit content of the element [46].

The concentration of calcium in the fruit varies. The highest levels of this element are present in the apple core. Then, its content decreases towards the outer part of the fruit, to rise again in the skin. As shown in various investigations, foliar Ca^2+^ fertilization was found to increase the concentration of this macronutrient in both the flesh and the skin of ‘Jonathan’, ‘Smoothee Golden Delicious’, and ‘Fuji/PAJAM1′ apples, with a significantly higher increase in the concentration in the peel than in the flesh [46,47]. Therefore, very high concentrations must be used to increase the calcium content in the fruit skin, although this has a negligible effect on the flesh. As a rule, these concentrations have a phytotoxic effect manifested by defoliation and reduction of yields. Given this fact, the use of lower calcium concentrations for reduction of bitter pit incidence in apples requires a choice of an appropriate surfactant to be added to the Ca solution. Ca^2+^ concentrations that are commonly recommended for spraying in orchards before harvest range from 0.03 to 0.1%, although some results show that this element is absorbed effectively when its concentration in the spraying solution is substantially higher, reaching 0.5%. Usually, several sprays are applied, as the concentration of Ca in the fruit decreases during the growing season. Information about Ca-spray programs was presented by Val et al. [46].

There are no data on the impact of foliar feeding with various forms of calcium on (i) the ultrastructure of apple fruit cells, (ii) qualitative and quantitative content of nutritional elements at the cell structure level, and (iii) distribution of Ca^2+^ cations in the cell wall and other parts of apple fruit cells at the cell level. Therefore, the aim of the study was to determine the effect of foliar feeding with calcium applied in mineral (Ca(NO_3_)_2_ and CaCl_2_) and organic-mineral (Ca chelated with EDTA) compounds on the ultrastructure of epidermis and hypodermis cells of *Malus domestica* Borkh ‘Szampion’. Additionally, a comparative microanalysis of the calcium content in selected parts of epidermis cells, and a mapping of the element, were carried out.

## 2. Results

### 2.1. Ultrastructure of Epidermis and Hypodermis Cells in Malus Domestica L. ‘Szampion’ Apples

#### 2.1.1. Epidermis

**Figure 1:**
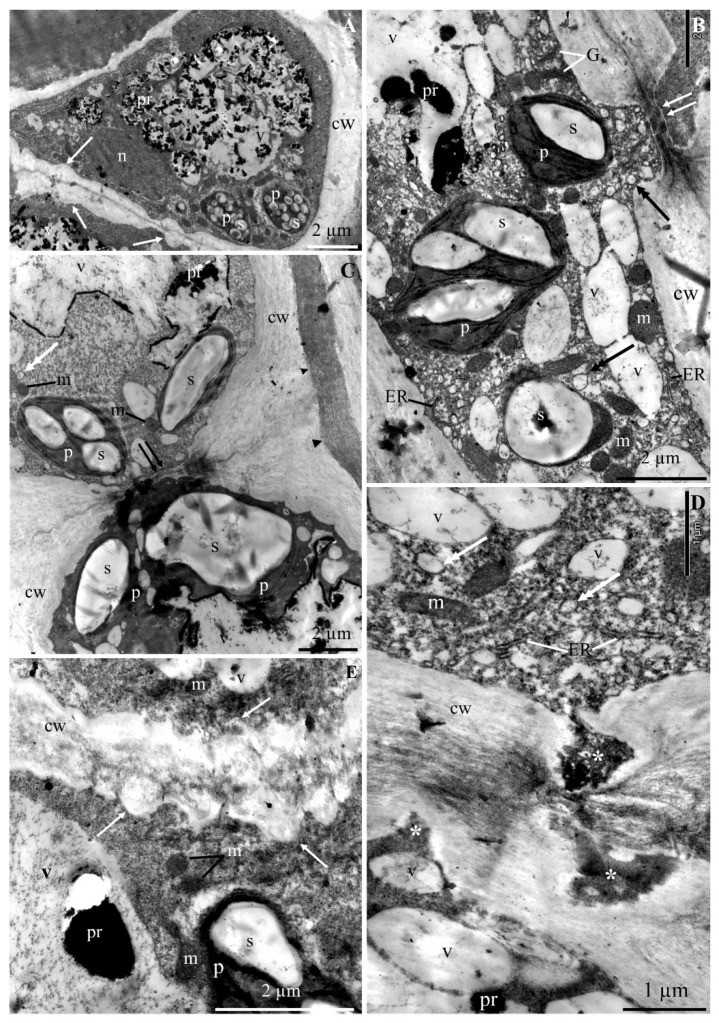
Fragments of the cross sections of epidermis and adjacent hypodermis cells from the apical part of ‘Szampion’ apples from the control treatment. **A**: epidermis cell, cell wall (cw) with invaginations (arrow), amyloplasts with starch grains (p), Golgi apparatus (G), cell nucleus (n), centrally located large vacuole (v) with dark material in cell sap (pr). **B**: epidermis cell with a visible cell wall (cw), middle lamella (two arrows), amyloplasts (p) with starch grains (s), mitochondria (m), transport vesicles (double-headed arrow), endoplasmic reticulum (ER), one large and several small vacuoles (v), precipitates (pr). **C**: hypodermis cells, visible cell wall with a dark-stained middle lamella (arrowhead), the middle lamella delineates the protoplasts of adjacent cells (two arrows), plastids (p) with starch grains (s), transport vesicles (double-headed arrow), discontinuous black-stained tonoplast (t). **D**: epidermis cell, visible cell wall discontinuity (cw), mitochondria (m), numerous transport vesicles (double-headed arrow), endoplasmic reticulum (ER), one large vacuole (v) or several small vacuoles with black-stained deposits (pr), cell wall evaginations (asterisk). **E**: epidermis and hypodermis cell wall (cw) with numerous invaginations (asterisk), mitochondria (m), plastids with starch grains (p), vacuole (v) with a fibrous substance and precipitate (pr).

**Figure 2:**
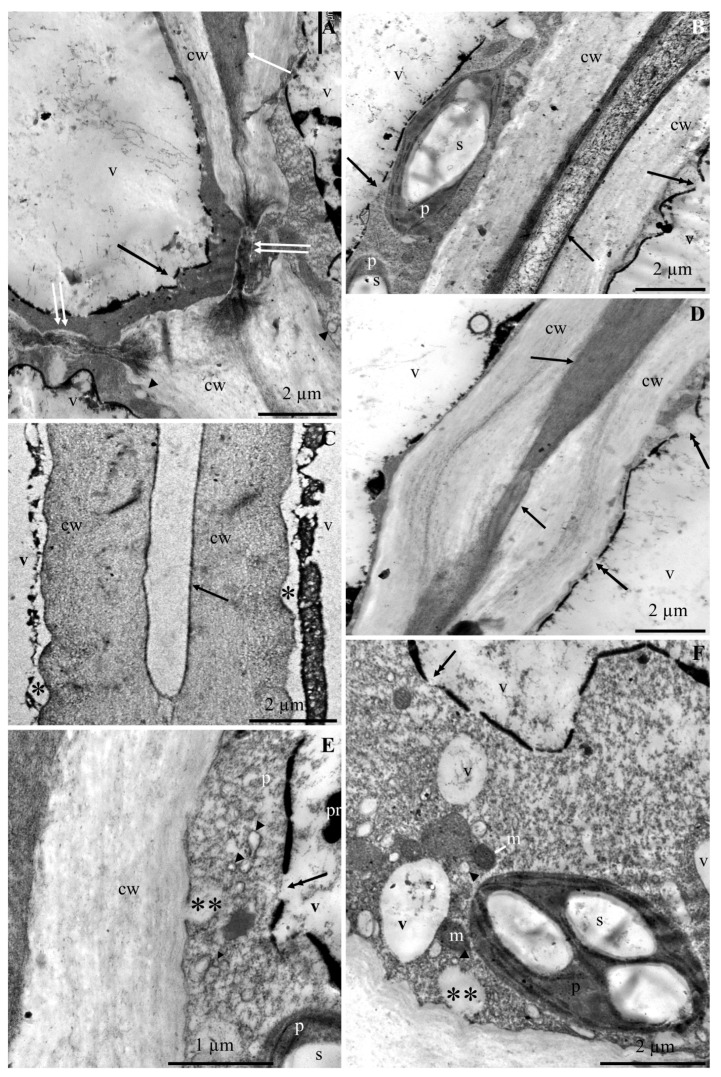
Fragments of the cross sections of hypodermis cells from the apical part of ‘Szampion’ apples from the control treatment. **A**: cell wall with varied thickness (cw), visible middle lamella (arrow), disappearance of the cell wall between the protoplasts of neighboring cells (two arrows), parietally located cytoplasm, discontinuous and intensively stained tonoplast (double-headed arrow), transport vesicles (arrowhead) near the plasmalemma, vacuole (v). **B**: cell wall (cw), visible middle lamella with loosened structure (arrow) (dissociation process), tonoplast discontinuity (double-headed arrow), plastids (p) with starch grains (s), tonoplast discontinuity (double-headed arrow), vacuole (v). **C**: dissociated cell walls (cw), disappearance of the middle lamella (arrow), periplasmic space between the cell wall and cytoplasm (asterisk), parietal cytoplasm, vacuole (v). **D**: cell wall (cw), varied thickness of the middle lamella (arrow), evaginated and loosened structure of the cell wall at the lamella narrowing, tonoplast discontinuity (double-headed arrow), vacuole (v). **E**,**F**: cell wall (cw), visible plasmalemma invaginations (two asterisks), tonoplast discontinuity (two arrows), different-sized transport vesicles (arrowhead), plastid (p) with starch grains (s), mitochondrion (m) (Figure 2F), tonoplast discontinuity (double-headed arrow), vacuole (v) with a flocculent substance in cell sap and a precipitate (pr) (Figure 2E).

**Figure 3:**
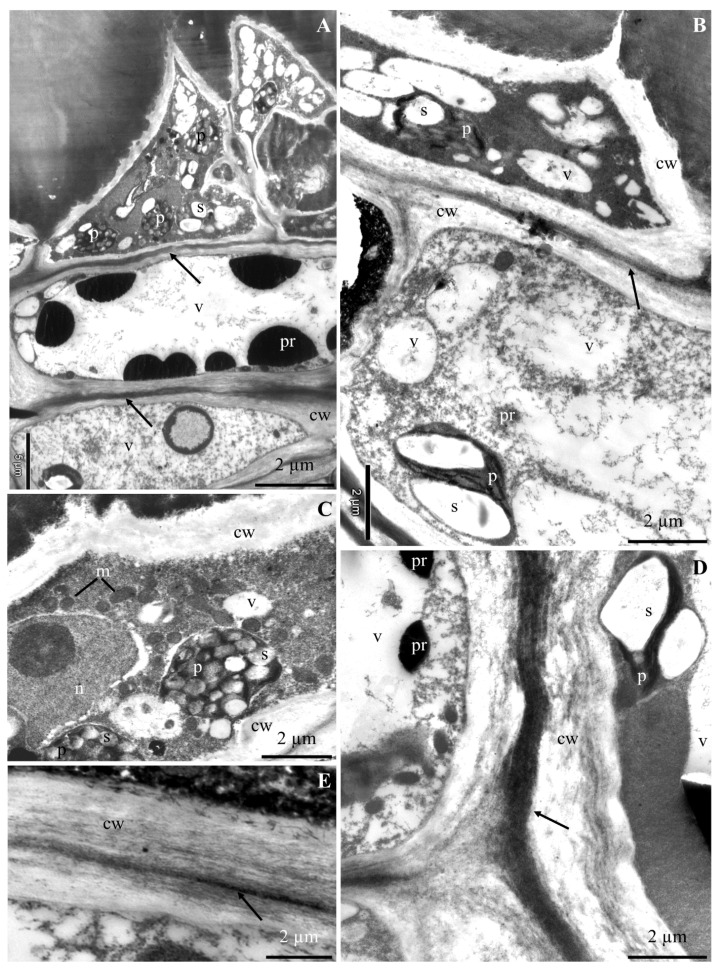
Fragments of the cross sections of epidermis and hypodermis cells from the apical part of ‘Szampion’ apples from the Ca(NO_3_)_2_ foliar feeding treatments. **A**,**B**: normally developed cell wall (cw) in epidermis and hypodermis cells, clearly visible middle lamella (arrow), plastids (p) with starch grains (s), vacuole (v) with precipitates (pr) adhering to the tonoplast and flocculent substance. **C**: epidermis cell wall (cw), numerous mitochondria (m), plastids (p) with starch grains (s), spherical cell nucleus (n) with a nucleolus, vacuole (v). **D**: hypodermis cell wall (cw), visible middle lamella (arrow), plastid with starch grains (s), vacuole (v), flocculent substance and precipitates (pr) in cell sap. **E**: hypodermis cell wall (cw), visible middle lamella (arrow).

**Figure 4:**
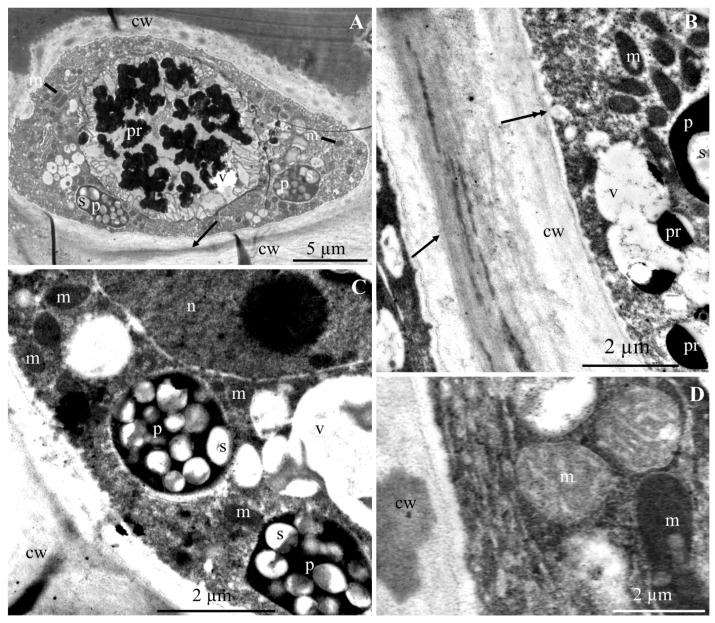
Fragments of the cross sections of epidermis cells from the apical part of ‘Szampion’ apples from the CaCl_2_ foliar feeding treatments. **A**,**B**: cell wall (cw), visible middle lamella (arrow), dense cytoplasm, plastids (p) with starch grains (s), numerous mitochondria (m), vesicular invaginations of the plasmalemma (double-headed arrow) (Figure 4B), vacuole (v), precipitates in cell sap (pr). **C**: protoplast, visible electron dense nucleoplasm of the cell nucleus (n) with a nucleolus, amyloplasts (p) with starch grains (s), mitochondria (m), vacuole (v). **D**: mitochondria (m) located near the plasmalemma, visible cell wall (cw) and electron dense cytoplasm.

**Figure 5:**
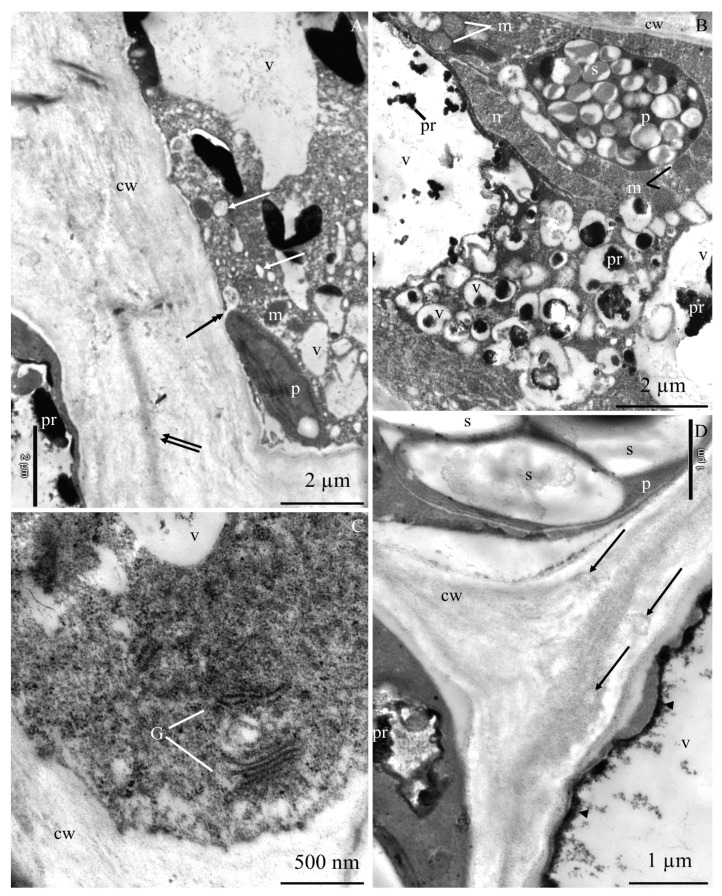
Fragments of the cross sections of hypodermis cells from the apical part of ‘Szampion’ apples from the CaCl_2_ foliar feeding treatments. **A**: cell wall (cw), visible middle lamella (two arrows), parietal cytoplasm with numerous transport vesicles (arrow), plasmalemma invaginations (double-headed arrow), plastid (p) with a starch grain (s). **B**: protoplast, visible lobular cell nucleus (n), large vacuole (v) and numerous small vacuoles, precipitates (pr) in cell sap. **C**: cell wall (cw), electron dense cytoplasm, Golgi apparatus (G), vacuole (v). **D**: cell wall (cw), visible vesicles (arrow), plastid (p) with starch grains (s), tonoplast (arrowhead), flocculent substance collected at the tonoplast and in cell sap.

**Figure 6:**
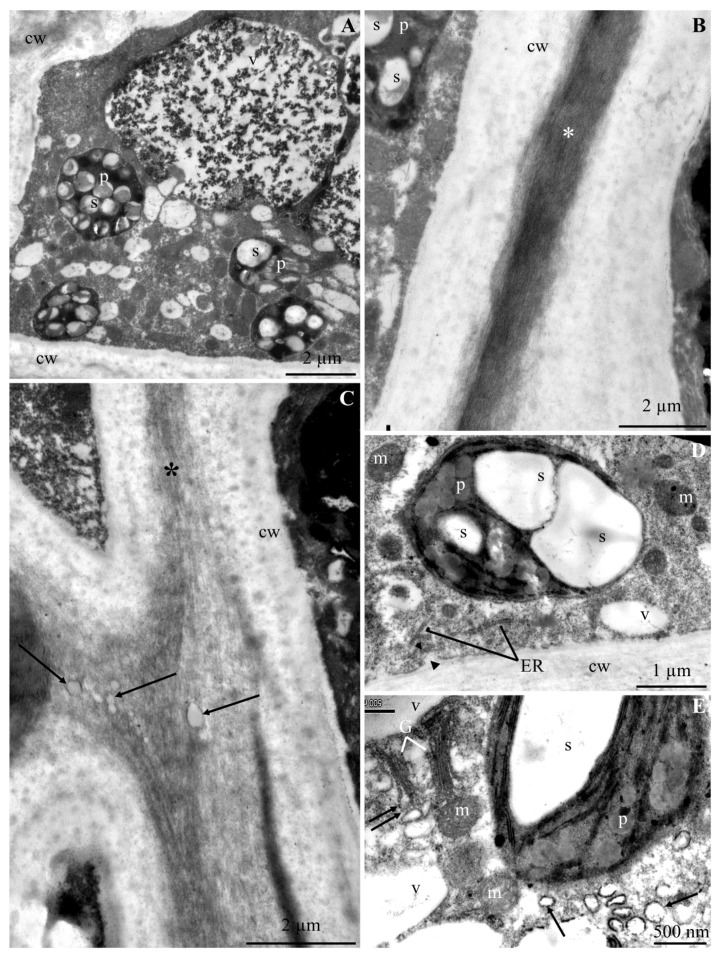
Fragments of the cross sections of epidermis cells from the apical part of ‘Szampion’ apples from the EDTA-chelated Ca foliar feeding treatments. **A**: cell wall, electron dense cytoplasm, plastids (p) with starch grains (s), vacuole (v), flocculent substance in the cell sap. **B**,**C**: cell wall (cw), visible middle lamella (asterisk), numerous transport vesicles in the cell wall (arrow) (Figure 6C), plastid (p) with starch grains (s) (Figure 6B). **D**,**E**: cell wall (cw) (Figure 6D), amyloplast (p) with starch grains (s), numerous mitochondria (m) located around the plastid, endoplasmic reticulum (ER) (Figure 6D), plasmalemma invagination (arrowhead) (Figure 6D), Golgi apparatus (G) with dictyosomal vesicles (two arrows) (Figure 6E), transport vesicles (arrow) (Figure 6E).

**Figure 7:**
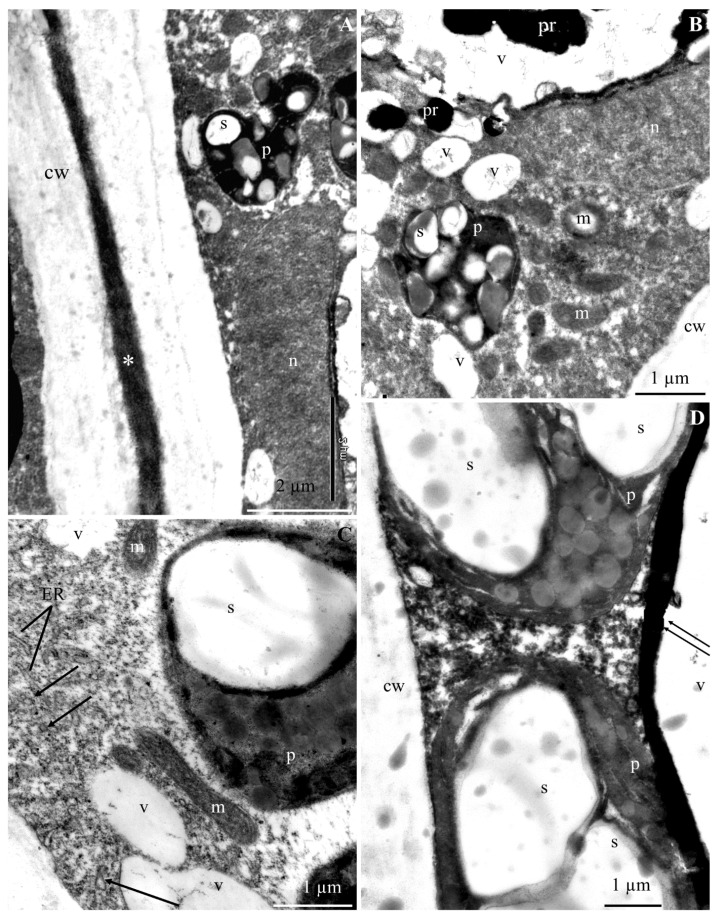
Fragments of the cross sections of hypodermis cells from the apical part of ‘Szampion’ apples from the EDTA-chelated Ca foliar feeding treatments **A**: cell wall (cw), visible middle lamella (asterisk), lobular cell nucleus (n), plastids (p) with starch grains (s). **B**,**C**: parietal cytoplasm, visible mitochondria (m), plastids with starch grains (s), large vacuole (v) or several small vacuoles, endoplasmic reticulum (ER) (Figure 7C); **D**: thick and intensely stained tonoplast (two arrows), visible cell wall (cw), plastids (p) with starch grains (s), vacuole (v).

The ultrastructure of the epidermis and hypodermis cells from the apical part of ‘Szampion’ apples was observed using a transmission electron microscope in the four experimental variants. The comparative studies revealed differences in the structure of the epidermis and hypodermis cells between the specimens from the control treatment (Figure 1A–E and Figure 2A–F) and those from plants receiving calcium foliar feeding (Figure 3A–E, Figure 4A–D, Figure 5A–D, Figure 6A–E and Figure 7A–D).

The cell wall in the epidermis and hypodermis cells in the control treatment had numerous evaginations (Figure 1A,E). In some fragments, the continuity of the cell walls was interrupted, and the middle lamella was visible (Figure 1B,C and Figure 2A). Near the middle lamella, there were numerous transport vesicles and plastids surrounded by mitochondria. The epidermis cell wall exhibited deep, dark-stained, irregularly shaped invaginations, which sometimes reached the middle lamella (Figure 1D).

The middle lamella had varied thickness (Figure 2A,D). Its fibrous structure was sometimes loosened or dispersed. Due to the destruction or partial disintegration of the fibrillar system, translucent empty spaces were visible in the central part of the lamella (Figure 2B). The middle lamella in some cells was expanded or narrowed. At the narrowing sites, the other part of the wall was evaginated (Figure 2D). The walls in some cells were completely dissociated. The periplasmic space was visible between the cell wall and the plasmalemma (Figure 2C). The plasmalemma formed different-sized vesicular invaginations (Figure 2E,F). The protoplast of the epidermis cells exhibited dense cytoplasm, a lobular nucleus, and numerous pleomorphic mitochondria located near plastids. The amyloplasts had one, two, three, or sometimes more starch grains. The cytoplasm contained transport vesicles, an endoplasmic reticulum, and one large or a few small vacuoles (Figure 1A–D).

In the variant with calcium nitrate foliar feeding, the epidermis cells had a normally developed cell wall and a more intensively stained middle lamella with a regular outline (Figure 3A,B,D). A similar wall structure was observed in specimens from the treatments treated with calcium chloride and Ca chelated with EDTA (Figure 4A,B and Figure 6B,C). The middle lamella contained vesicles (Figure 5D and Figure 6C). As in the control treatment, the plasmalemma had numerous vesicular invaginations towards the protoplast (Figure 4B). There were numerous transport vesicles and ER near the plasmalemma. The cytoplasm contained a spherical or lobular cell nucleus (Figure 3C and Figure 4C), plastids with starch grains, numerous mitochondria located close to amyloplasts (Figure 3A–C and Figure 4A–D), Golgi apparatus, and dictyosomal vesicles (Figure 6E). The tonoplast in the apples from the three foliar feeding treatments retained its continuity (Figure 3B, Figure 4A–C and Figure 6A). A dark precipitate was detected in cell sap in small or large vacuoles (Figure 3B, Figure 4A and Figure 6A).

#### 2.1.2. Hypodermis

The hypodermis cell wall in the fruit sections from the control treatment is described in the subsection on epidermis. The cytoplasm in the hypodermis cells was located parietally. The cytoplasm contained amyloplasts with starch grains, mitochondria, and numerous vesicles (Figure 1C,E,F and Figure 2B,E,F). The distinct tonoplast was dark and discontinuous (Figure 1C and Figure 2A,B,D–F). A flocculent substance in the cell sap contained in a large centrally located vacuole was visible near the tonoplast (Figure 1C and Figure 2A,B,D–F).

The walls in the hypodermis cells in the samples from the calcium foliar feeding treatments were formed normally (Figure 3A,B,D,E, Figure 5A,D and Figure 7A). Different-sized vesicles were present in the cell walls (Figure 5D). The plasmalemma adhered to the cell wall and there were visible membrane invaginations (Figure 5A). Transport vesicles were located next to the plasmalemma (Figure 5A and Figure 7C). The cytoplasm formed a thin parietal layer and contained amyloplasts (Figure 3B,D, Figure 5B and Figure 7A–D), Golgi apparatus (Figure 5C), and ER (Figure 7C) as well as numerous mitochondria located close to plasmalemma or plastids (Figure 3C, Figure 5A,B and Figure 7B,C). The tonoplast sometimes had a thick outline and was intensely stained (Figure 5D and Figure 7B,D). A flocculent precipitate or semicircular black crystals accumulated near the tonoplast (Figure 3A,B,D, Figure 5A,B and Figure 7B). The cell sap contained a flocculent substance or numerous black-stained precipitates (Figure 3A,B, Figure 5A,B,D and Figure 7B).

Summarizing, the cell wall in the epidermis and hypodermis cells of fruit samples from the combined foliar feeding treatments was properly formed and had a clearly marked middle lamella. The cytoplasmic membranes retained their continuity. Numerous transport vesicles, with greater abundance close to the plasmalemma, were observed in the cytoplasm. In turn, in the Ca^2+^ nonsupplemented variants, most of these cells exhibited wall deformations such as a loosened fibrillar structure of the middle lamella resulting in wall dissociation and different sized invaginations in the other parts of the wall, resulting in their discontinuity and rupture of the protein-lipid membranes.

### 2.2. Calcium Content at the Epidermis Ultrastructure Level

#### 2.2.1. Calcium Content in Selected Cell Elements

**Figure 8:**
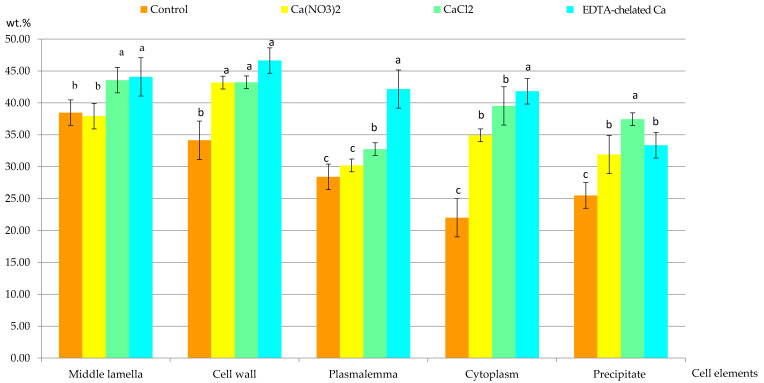
Content of Ca^2+^ ions in selected epidermis cell elements and precipitates (calcium oxalate crystals) in the apical part of ‘Szampion’ apples. Means (±SD) for each of cell element marked with different letter above the bars are significantly different at *p* ≤ 0.05.

The content of Ca^2+^ cations in the selected elements of epidermis cells from the apical part of the apples in the control and calcium foliar feeding treatments varied. At the cellular level, the highest concentration of these ions was detected in the middle lamella, other parts of the cell wall, the plasmalemma, and the cytoplasm in each combination of the experiment. The concentration of Ca^2+^ cations in the middle lamella was 38.5 wt.% in the control treatment and from 38 (Ca(NO_3_)_2_ to 44 wt.% (Ca chelated with EDTA) in the foliar feeding variants. The content of this element in the cell wall was 34 wt.% in the control and from 43 to 46 wt.% in the foliar feeding treatments. The lowest Ca^2+^ concentration was detected in the plasmalemma of epidermis cells in the control treatment. It was slightly higher in the samples from the variants treated with calcium nitrate and calcium chloride and significantly higher in the Ca chelated with EDTA) supplemented apples. The following order of treatments with increasing concentrations of Ca^2+^ ions in the cytoplasm was established (wt.%): control (30.2) < Ca(NO_3_)_2_ (34.9) < CaCl_2_ (39.5) < Ca chelated with EDTA) (41.8). In turn, the Ca^2+^ concentration in the crystalline dark-stained precipitates present in the cell sap was in the range from 32 (control) to 37 wt.% (CaCl_2_). The concentration of Ca^2+^ ions in all the elements of epidermal cells analyzed suggests that, generally, the Ca foliar feeding significantly increased the content of this element regardless of the form of the compound. The highest increase in the content of Ca^2+^ cations was found in the combination of Ca chelated with EDTA. In this variant, compared to the control treatment, the concentration of Ca^2+^ cations in the middle lamella, other elements of the wall, plasmalemma, and cytoplasm increased by approximately 1.15; 1.34; 1.49, and 1.89 times, respectively (Figure 8).

#### 2.2.2. Calcium Content in Selected Fragments of Epidermis Cells

**Figure 9:**
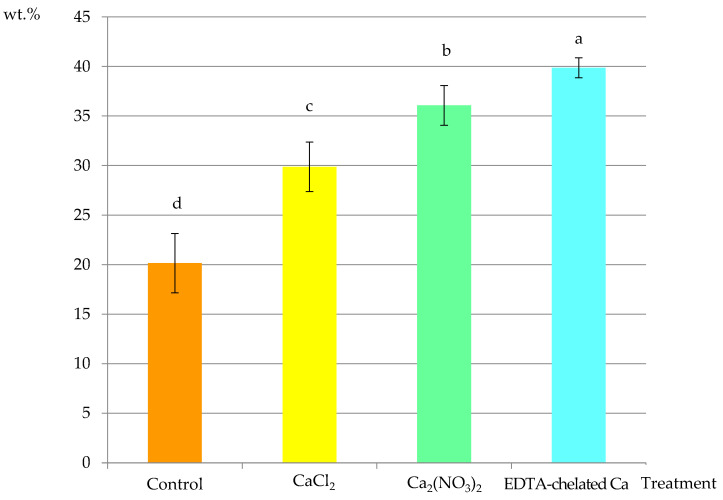
Ca^2+^ content in fragments of epidermis cells from the apical part of ‘Szampion’ fruits in the control treatment and Ca foliar feeding variants. Means (±SD) marked with different letter above the bars are significantly different at *p* ≤ 0.05.

The comparative analyses of fragments of epidermis cells, i.e., the cell wall, plasmalemma, and cytoplasm, demonstrated 20 wt.% content of Ca^2+^ ions in the control treatment. Apples supplemented with Ca(NO_3_)_2_, CaCl_2_, and Ca chelated with EDTA exhibited calcium concentrations of 30 wt.%, 37 wt.%, and 39 wt.%, respectively. In comparison to the control, the content of this element increased by 50%, 82%, and 93%, respectively. In the Ca-supplemented variants, there were significant differences between the Ca(NO_3_)_2_ and EDTA-chelated Ca treatments. These values indicate that the EDTA-chelated Ca variant was the most effective treatment in increasing the concentration of Ca^2+^ cations in the epidermis cells (Figure 9).

### 2.3. Ca^2+^ Mapping at the Level of Epidermis Cell Ultrastructure

**Figure 10:**
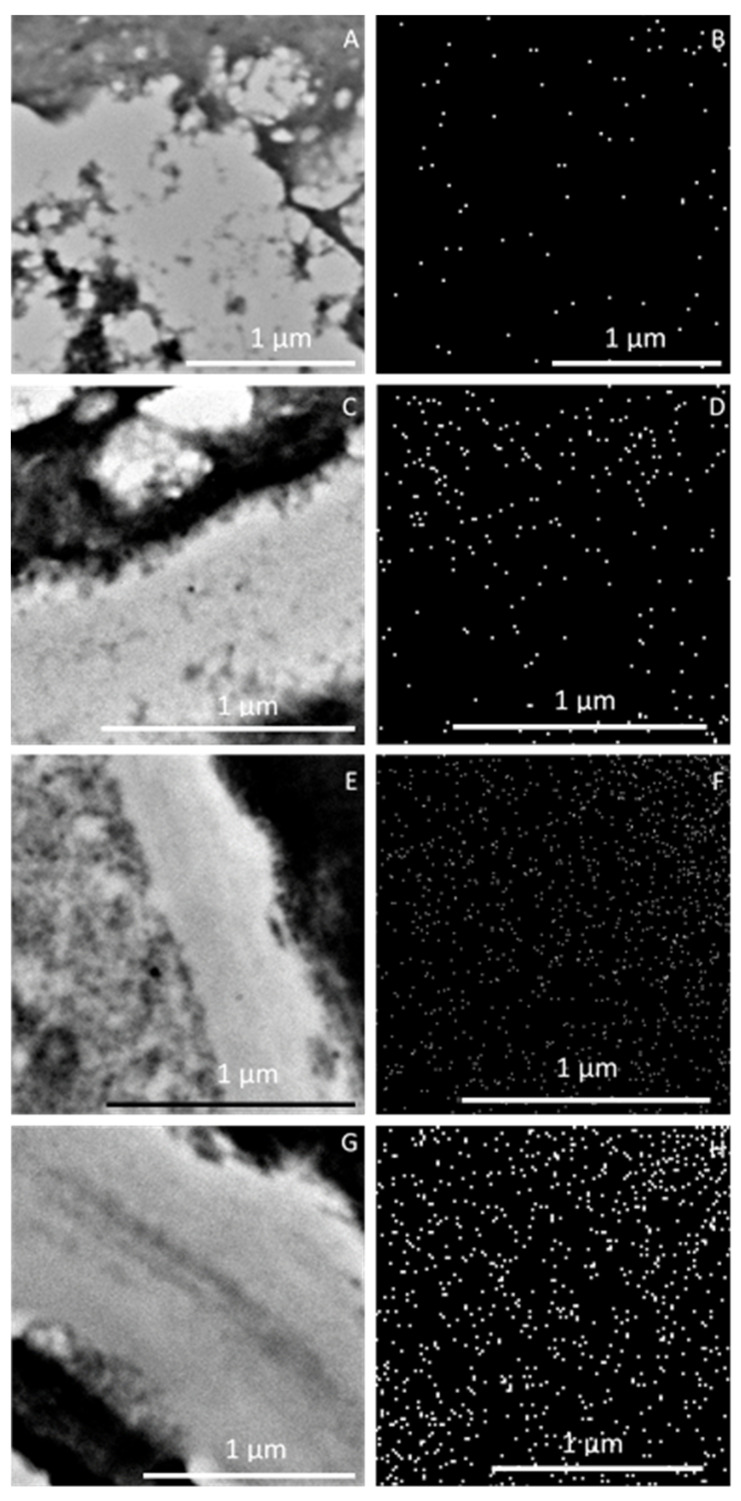
**A**–**H**. Fragments of epidermis cells (**A**,**C**,**E**,**G**) and mapping Ca^2+^ cations in the section (**B**,**D**,**F**,**H**) including the cell wall, plasmalemma, and cytoplasm from the apical part of ‘Szampion’ apples from the control treatment (**A**,**B**) and the Ca(NO_3_)_2_ (**C**,**D**), CaCl_2_ (**E**,**F**), and EDTA-chelated Ca (**G**,**H**) foliar feeding variants. A,C,E,G—TEM, B,D,F,H—EDS INCA Energy TEM.

The Ca^2+^ cation mapping revealed varied distribution of this macronutrient in the analyzed fragments of epidermal cells, i.e., the cell wall, plasmalemma, and cytoplasm (Figure 7A,C,E,G). It was shown that, in comparison with the control treatment (Figure 7b), the frequency of Ca^2+^ ions in the foliar feeding variants increased (Figure 10D,F,H), especially in the third—CaCl_2_ (Figure 10F)—and fourth—EDTA-chelated Ca (Figure 10H)—treatments.

## 3. Discussion

### 3.1. Impact of Ca^2+^ Cations on the Cell Ultrastructure

The analyses showed numerous invaginations and evaginations of the cell wall in the control treatment compared to the calcium-supplemented apples. The fibrous structure of the middle lamella was loosened, and its fibrillar system was sometimes completely disintegrated. This destruction resulted in dissociation of the cell walls. Cell wall thickening was observed in addition to the disappearance of the middle lamella. As suggested in literature reports, the disturbances in the wall structure detected in the present study result from calcium deficiency, as this macronutrient is responsible for stabilization of cell walls and activity of enzymes in this part of the cell. Calcium is involved in the formation of bridges between cellulose and pectin micelles contributing to enhancement of cell wall stiffness. It is postulated that pectins exert two effects on the cell wall: they increase the influence of calcium on mechanical parameters and counteract the stretching force [48]. Ca^2+^ cations have a substantial effect on the density of non-dispersed charges, and thus play a key role the function of the cell wall [49]. This element is responsible for maintenance of the structure of the cell wall, especially the middle lamella [50]. Thickening of the cell wall and dissolution of the middle lamella accompanying Ca^2+^ deficiency were observed in stored apples by other researchers [51]. Calcium-bound pectins are highly important for the spatial structure and mechanical strength of cell walls [52].

The present study showed reduction of cell wall destruction upon application of calcium through foliar feeding. The wall structure was normal with a regular middle lamella. Numerous vesicles were present in the fibrillar system of the walls. Similarly, other authors reported that additional calcium application exerted a positive effect on the cell wall. As shown by Glenn and Poovaiah [51], the cell wall stability and elasticity were positively correlated with the Ca content in the hypoderm. Additionally, these authors found that the presence of Ca^2+^ cations inhibited the solubilization of polyuronide and arabinose molecules and increased the galactose content during apple fruit storage.

A periplasmic space between the cell wall and plasmalemma in the control samples was shown in the present investigations. The membrane exhibited evident invaginations. A detached protoplast suggests disturbances in water metabolism manifested by plasmolysis. In turn, this process did not occur in the cells of apples from the calcium foliar feeding variants. This indicates that calcium has a protective effect on the structural integrity and fluidity of cytoplasmic membranes. This protective effect is associated with the fundamental role of calcium in processes determining the normal architecture/composition and function of membranes and with the involvement of Ca^2+^ in the control of the activity of wall enzymes and stabilization of cell wall structures. All these properties originate from the tight Ca^2+^ interaction with phospholipids and proteins [53]. Due to its high affinity, calcium significantly affects the density of non-diffusible charges, which is the most important factor in the control of the wall behavior. In addition, calcium is involved in signal transduction in plants [16,54,55,56,57]. Hormonal signaling is one of the physiological mechanisms related to the signs of the deficiency of this element in apples (bitter pit) [58]. The binding of Ca^2+^ to Ca^2+^-sensors, such as calmodulin, induces appropriate physiological cellular responses via modulation of the functions of target proteins, especially kinases involved in a plethora of cellular processes, including ion transport, metabolism, post-translational protein modifications, and gene expression [59,60]. Ca^2+^ activates the SOS3-SOS2 protein kinase pathway, which leads to enhanced expression of the plasma membrane Na^+^ efflux transporter (SOS1) and restricts Na^+^ influx through the plasma membrane high-affinity K^+^ transporter (HKT1), helping to maintain intracellular K^+^/Na^+^ homeostasis [61]. Through its impact on colloids, calcium enhances the viscosity and reduced the hydrophilicity of the cytoplasm, which is highly important for permeability of cell membranes [16].

In addition to stabilization of membranes, calcium modifies the properties of the cell wall through crosslinking of de-esterified pectins. Apoplasmic calcium–pectin crosslinking, mainly in the xylem, exerts a significant effect on the fruit water relations. Normal distribution and accumulation of calcium in fruit depends on water metabolism and cell wall interactions in the apoplast [56,57]. Quiles et al. [62] reported that calcium feeding strengthened the structure of cell walls, prevented cell collapse, and protected protoplasts from plasmolysis.

In the cells of the control apples, the tonoplast was discontinuous in many areas. As demonstrated by literature data, discontinuity of some plasma membranes can be caused by both deficient and excessive calcium concentrations, which can damage the membranes [63]. Fukumoto and Venis [64] demonstrate that the tonoplast in apples has a calmodulin-dependent active Ca^2+^ transport system. These membranes usually retained continuity in the calcium foliar feeding treatments in the present study. In many cells, the tonoplast had a larger outline. In other investigations, the cells of calcium-treated apples had a more compact, stronger, and thicker wall, plasmalemma, and tonoplast [65]. As shown by Wang et al. [63], tonoplast discontinuity resulted in modification of its selectivity without complete destruction of the vacuole. Investigations conducted by Mauro et al. [66] prove that calcium prolongs cell viability and increases the effectiveness of osmodehydration.

In the present study, a flocculent precipitate was detected in the sap juice at the tonoplast of the calcium-fed apples. Black-stained precipitates (calcium oxalate crystals) were visible in the large and smaller vacuoles in samples from all experimental treatments. These crystals are commonly found in many taxonomic units. They can have different shapes and sizes. Their main function in plants is regulation of the Ca^2+^ concentration and protection against herbivores. Biomineralization is a physicochemical process of precipitation of endogenous oxalic acid and exogenous Ca^2+^ cations [67,68,69,70]. The morphology of these crystals within a species is invariable and subject to genetic regulation. There is also a close relationship between crystal growth and cell expansion [67].

The present comparative studies conducted with the use of light and transmission electron microscopy have shown the presence of an increased number of amyloplasts in the epidermis and hypodermis cells in samples from the calcium foliar feeding variants, in comparison with the control treatment. The greater number of amyloplasts in the cells from apples treated with the Ca foliar feeding indicates an increased concentration of starch. As demonstrated by literature data, Ca^2+^ cations may cause changes in the tertiary structure of α-amylase, i.e., an enzyme degrading starch accumulated in amyloplasts [71]. In turn, the presence of micromolar amounts of calcium is required for stabilization of the structure of this enzyme. The importance of calcium in starch hydrolysis depends on the structure of starch substrates [72].

The ultrastructural changes detected in the present study were accompanied by differences in the values of harvest maturity indicators and the nutritional fruit value. In the calcium-supplemented combinations, regardless of the form applied (Ca(NO_3_)_2_; CaCl_2;_ Ca chelated with EDTA), higher fruit firmness and higher total sugar content post-harvest and after 5-months storage were found as well as a significant increase in the vitamin C content in the Ca(NO_3_)_2_ treatment_,_ compared to the control. The firmness of the flesh is an important parameter of the quality of apples. In most varieties, this parameter is correlated with the level of calcium. Hence, calcium supplementation treatments constitute an important standard element of production technology. Many authors [40,47,73] have reported changes in fruit quality traits, particularly flesh firmness, acids, and colour, associated with increases in the Ca concentration in the fruit flesh following the application of several Ca-sprays. In turn, Val et al. [46] did not show a significant effect of calcium chloride spraying on these parameters, which, as suggested by the authors, resulted from the slight changes in the Ca concentration in the pulp.

The present observations indicate that the disturbances in the ultrastructure of the fruit cells of the calcium non-supplemented control were manifested by mildly intensified symptoms of bitter pit. In contrast, the other calcium-supplemented combinations characterized by normal ultrastructure of cells exhibited no symptoms of this physiological disease during the harvest and storage periods. Improvement of fruit quality after foliar spray with 0.3% CaCl_2_ has been reported by Rab and Haq, [74], who found not only a rise in the number of fruit but also a decrease in the incidence of blossom end rot in tomato (*Solanum lycopersicum*). Furthermore, foliar sprays of CaCl_2_ reduced postharvest decay and improved firmness retention in sweet bell pepper (*Capsicum annuum*) [75]. Similarly, calcium nitrate foliar application on salt-treated lettuce (*Lactuca sativa*) and endive (*Cichorium endivia*) did not affect plant growth but reduced symptoms of physiological diseases, e.g., blackheart and tip-burn [76].

### 3.2. Content of Ca^2+^ Cations at the Cellular Level

Increased content of an element in plant biomass is a typical response to an increase in its concentration in the plant nutrient environment, and this regularity has been noted for both nutrients and trace elements. The increase in the content of Ca^2+^ cations in the selected fragments of epidermis cells from the apical fruit region, visualized by mapping thereof, undoubtedly results from the function of this macronutrient as a cell binder (calcium is a component of the middle lamella) and its role in the formation, structure, and regulation of the function of cell walls and membranes. The chemical form of the Ca compound is one of the determinants of the effectiveness of Ca spray treatments [77,78].

The calcium preparations used in the presented study significantly increased the concentration of Ca^2+^ ions in the elements of the fruit epidermis cells. This was confirmed by the analysis of Ca^2+^ ions in the individual fragments of the cells in this tissue. Calcium applied in the organic-mineral form (Ca chelated with EDTA) penetrated into the fruit cells much more easily than when applied in the mineral forms (calcium chloride and calcium nitrate). This indicates that the organic compound accompanying Ca^2+^ ions overcomes barriers against the entrance of exogenous substances much more easily than calcium in mineral compounds, which results from the presence of accompanying ions (Cl^−^, NO_3_^−^) and structural differences.

Chelated fertilizers, i.e., complex compounds in which an organic ligand is coupled with a central ion via more than one coordination bonds, are considered to be the most effective form of supplementation of plants with nutrients. The chelate form ensures excellent miscibility and water solubility, as well as stability over a wide pH range. Due to the tight bonds, chelated compounds have considerably higher stability than non-chelated ones. Chelated fertilizers easily penetrate protein-lipid membranes and chelates taken up as whole particles by roots or leaves are translocated in plants as quickly as metal ions [79]. Calcium nitrate contains 15.5% of nitrogen in its composition. Due to the high demand for nitrogen, plants take up substantial amounts of nitrates in the form of NO_3_^−^ anions, additional amounts of Ca^2+^ cations to maintain the ionic balance. In turn, calcium chloride, unlike calcium nitrate, is a physiologically acidic salt from which calcium is taken up by plants more readily than chlorine [80]. Ca(NO_3_)_2_ has the potential to increase plant growth and divert Ca away from the xylem; for this reason, CaCl_2_ is selected as the foliar source in field experiments. Calcium penetration through the fruit epidermis is of key importance for the effectiveness of Ca^2+^ spray treatments [46]. The penetration of chemicals largely depends on the point of deliquescence of salts [81]. In this sense, CaCl_2_ has a lower value of this parameter than other Ca salts, for which its effectiveness is greater.

However, as indicated by the results obtained in the present experiment, CaCl_2_ proved to be a more effective source of calcium than Ca(NO_3_)_2_, which was evidenced by the higher weight percentage of Ca^2+^ in the analyzed cell constituents (middle lamella, cell wall, plasmalemma, cytoplasm) after the application of the former nitrogen form. There are many literature data showing an increase in calcium content as a result of foliar feeding of plants with Ca(NO_3_)_2_ and CaCl_2_. For example, Chondraki et al. [82] revealed that foliar application of Ca(NO_3_)_2_ increased leaf Ca content in parsley [*Petroselinum crispum*]. Mayr and Schröder [83] showed that the treatment with 10 kg of calcium chloride in 500 liters per hectare sprayed every 2 weeks after June drop until harvest increased the calcium content in ‘Boskoop’ or ‘Elstar’ apples on average by 29% or 18%, respectively. Nearly the same effect was found for the pretreatment with prohexadione-Ca. In contrast, Qiu et al. [84] did not find the increase in Ca concentrations in ‘Kapoho Solo’ papayas grown in Hawaii after application of CaCl_2_ sprays. Similarly, Madani et al. [85] did not report changes in calcium concentrations induced by preharvest foliar sprays with three different sources of Ca (CaCl_2_, Ca(NO_3_)_2_, and Ca(C_2_H_5_COO)_2_) at four concentrations: 0, 60, 120, and 180 mg per litre.

There is abundant evidence that the penetration rates of CaCl_2_ in apples are affected by the fruit development stage, i.e., the penetration rate is higher in early fruit development stages and decreases rapidly in later stages [86,87]. Schlegel and Schonherr [87] reported that rapid penetration of Ca from foliar application into young apples occurred through trichomes and stomata until day 45 after full bloom, when the fruit shed trichomes and penetration continued mainly via lenticels. Neilsen et al. [40] indicated comparable effectiveness of CaCl_2_ spray in the early and late growing season, despite the low Ca concentration in the harvested fruit. As demonstrated by Shiri et al. [88], the absorbability of Ca by kiwifruit ‘Hayward’ from Ca spray (CaCl_2_, 15 g per liter) decreased significantly with the progressing fruit development, and the highest fruit Ca content was found when the fruit plants were sprayed at days 35 + 80 after full bloom and days 35 + 80 + 120 after full bloom, irrespective of the location of the orchard. They also found that CaCl_2_ sprays effectively improved the fruit quality by balancing the Ca ratio to N, K, and Mg.

Literature data indicate that dysfunction of the cell wall and disturbances in the membrane system are the two main targets of the effect of low Ca^2+^ concentrations. In calcium deficit, membrane permeability increases, leading to the efflux of ions and metabolites. In turn, Ca^2+^ ions in the cell walls play a key role in pectin crosslinking [16,89,90]. During the formation of the cell wall, acidic pectin residues (mainly galacturonic acid) are released as methyl and de-esterified residues with simultaneous release of Ca^2+^ binding carboxylic groups. Therefore, a low intracellular calcium level contributes to an increase in elasticity and facilitates the rupture of the cell wall, whereas high Ca^2+^ concentrations reinforce the wall and reduce its plasticity. Calcium is indispensable for structural integrity and maintenance of selectivity of cell membranes. It has been demonstrated that the level of intracellular calcium determines the integrity of cell membranes by stabilization of two lipid layers via interactions with phospholipids [16].

In turn, the cytoplasmic calcium level is crucial for the mechanisms of both short- and long-distance signaling through the allosteric regulatory effect on many enzymes and proteins. This is especially important in defense reactions to various types of abiotic and biotic stresses. Calcium is well known to act as a secondary transmitter of signals produced by indirect transduction pathways [91]. Such signaling is possible due to changes in the protein conformation induced by Ca^2+^ ions and mechanisms controlling the level of calcium in the cytoplasm and organelles [92,93,94], in contrast to other transmitters, whose level in the cell is regulated by synthesis and decomposition. The concentration of calcium ions in the cytoplasm is a consequence of their controlled influx mainly from the endoplasmic reticulum and from the external environment. Due to the activity of membrane calcium pumps, which continuously pump out calcium ions from the cytoplasm, it is possible to maintain a substantially higher concentration of Ca^2+^ ions outside the cell than in the cytoplasm. The controlled Ca^2+^ cation influx through the cell membrane proceeds via calcium channels [95,96,97]. Mitochondria located in the immediate vicinity of the endoplasmic reticulum and proteins prevent an uncontrolled increase in Ca^2+^ concentrations in the cytoplasm [98]. A relatively constant level of calcium ions in the cytoplasm facilitates efficient transmission of signals from the environment to the cell and ensures a normal course of calcium-controlled processes. Besides its function as a secondary transmitter, calcium limits the effects of various types of stress by neutralization of reactive oxygen species induced in adverse conditions [99,100,101].

Calcium also regulates the activity of many enzymes [102,103]. It inhibits the activity of potassium-dependent enzymes, exerting an indirect impact on the respiration and energy metabolism [104,105,106]. It has been proven that the root application of conventional calcium fertilizers does not significantly influence calcium transport in the plant, and an increase in their doses does not always result in enhanced fertilization efficiency [78,107].

## 4. Material and Research Methods

### 4.1. Study Area and Experimental Treatments

The investigations were conducted using apple trees (*Malus domestica* L. ‘Szampion’). The experiment was carried out in a commercial orchard in Piotrowice Małe, Nałęczów Commune (Piotrowice Małe, Poland) (51°18′39″ N, 22°14′15″ E), in 2011–2013. The trees grew on sandy loam, with the following particle size distribution: (a) arable layer 0–20 cm, fractions: sand 0.05–2.0 mm—52%, silt 0.002–0.05 mm—42%, clay 0.002 mm—6%, (b) subsoil layer 20–40 cm, fractions: sand 0.05–2.0 mm—52%, silt 0.002–0.05 mm—41%, clay 0.002 mm—7%.

Five-year-old trees were grafted on rootstock M.26. They were planted in 2008 in a row-and-belt system with 4 × 1.5 × 1m spacing. The density was 2160 trees per hectare. The tree rows were located on a herbicide fallow strip, and the inter-rows were covered by grass. A one-factor design was used in the vegetation experiment. It was based on a completely randomized block design in four repetitions. A 30-m^2^ plot with five trees was the experimental unit. The experiment consisted of applications of various formulations containing calcium, in a mineral and chelated form, in four study treatments, including the control with no additional Ca supplementation (I) and foliar applications of Ca(NO_3_)_2_—15.5% N and 19% Ca (II), water-soluble highly hygroscopic calcium chloride CaCl_2_—63.6% Cl and 36.4% Ca (III), and Ca chelated with EDTA (ethylenediaminetetraacetic acid)—9.5% Ca (IV). The trees in the control treatment were sprayed on the same date with pure water used to prepare the working solution.

In each study year, calcium foliar feeding was initiated during the last 10 days of June, when the apples were in the “walnut” phase and continued until mid-September in the harvest maturity phase. The treatments were applied in the late afternoon on windless and rainless days every second week, i.e., the trees were fertilized seven times per season, in accordance with foliar feeding recommendations. The concentration of the working solution was 0.50% Ca(NO_3_)_2_, 0.27% CaCl_2_, and 0.25% EDTA Ca. The concentration of calcium in the working solution was 1000 mg Ca liter, whereas the concentration of the chelated form was consistent with the manufacturer’s instructions (2.0 kg ha^−1^). The working solution was applied with a sprayer until the entire tree crown was wetted. The amount of the liquid used was 800 liters per hectare.

### 4.2. Plant Material

Apples intended for sampling were harvested in mid-August from 10 randomly selected trees in each experimental variant. Two apples were taken from each tree following the same pattern (southern side of the tree, comparable location in the crown, similar fruit diameter). Two fragments (5 × 5 mm) with epidermis, hypodermis, and adjacent parenchyma cells were excised from the apical part of each fruit. The samples were fixed for comparative observations of cell ultrastructure, and the microanalysis and mapping of Ca^2+^ cations.

### 4.3. Fixation of Plant Samples

Fragments collected from the apical parts of the apples were fixed in a 4% glutaraldehyde solution in 0.1 M phosphate buffer, pH 7.0, at room temperature for 6 h. The samples were rinsed in 0.01 M phosphate buffer, pH 7.0, at 4 °C for 48 h and contrasted in a 1.5% aqueous osmium tetraoxide solution for 1.5 h (samples for TEM observations). Fragments intended for the microanalysis and Ca^2+^ cation mapping were not treated with osmium. After rinsing the sections with distilled water, a 0.5% aqueous uranyl acetate solution was applied at room temperature for 2 h. After another two washes with distilled water, the plant material was dehydrated in a series of ethyl alcohol at the successive concentrations of 15, 30, 50, 70, 90, 96%, and 99.8% (anhydrous ethanol was used twice) for 15 min in each solution. The dehydrated plant samples were embedded in Spurr Low Viscosity resin and polymerized at 60 °C for 48 h.

### 4.4. Microscopic Study Methods

The analysis of the structure of epidermis and hypodermis cells consisted in (i) anatomical and (ii) ultrastructural observations of epidermis and hypodermis cells, (iii) microanalysis of the calcium content in the outer periclinal cell wall, plasmalemma, cytoplasm, and precipitates, and (iv) Ca^2+^ mapping with the use of bright-field microscopy (LM, Tokyo, Japan), transmission electron microscopy (TEM, Hillsboro, OR, USA), and an X-ray microanalyzer (MR, Oxford, UK).

#### 4.4.1. Bright-Field Microscopy

After preparation of blocks, 1-μm thick sections were cut with a glass knife using a Reichert Ultracut S microtome (Wetzlar, Germany). Fruit fragments were stained with 1% toluidine blue with 1% azure II (1:1) at 60 °C for 5 min. The semi-thin sections were rinsed with distilled water and 5% ethyl alcohol and embedded in Eukitt after drying. The semi-thin cross-sections prepared from the fixed material were analyzed using a Nikon Eclipse 90i bright field microscope (Tokyo, Japan). Based on the observations of the anatomical structure of epidermis, hypodermis, and parenchyma cells in the samples from the four experimental combinations, fragments of sections were selected for the comparative ultrastructural studies.

#### 4.4.2. Transmission Electron Microscopy

Ultrathin 70-nm sections were cut from the fixed and resin-embedded apical fruit sections for TEM observations. The fragments were stained with an 8% uranyl acetate solution in 0.5% acetic acid for 40 min and rinsed twice with distilled water (10 min). Next, they were contrasted with the Reynolds Reagent for 15 min [108]. The sections were rinsed with water and dried. Observations of the ultrastructure of epidermis and hypodermis cells were carried out using a FEI Tecnai Spirit G^2^ transmission electron microscope (TEM) (Hillsboro, OR, USA).

### 4.5. Microanalysis of Ca^2+^ Cations at the Cell Ultrastructure Level

The microanalysis of the calcium content in the outer anticlinal cell wall, middle lamella, plasmalemma, cytoplasm, and precipitates in the epidermis cells from apples sampled from the four experimental treatments was carried out using ultrathin non-contrasted sections (100 nm thick). In each experimental combination, 4–5 sections were analyzed, choosing appropriate areas for measurements of the Ca^2+^ content (n = 12). The microanalysis of calcium was performed with a high-resolution transmission electron microscope JEM 1400 (JEOL Co., Tokyo, Japan 2008) and a digital microscope coupled with the Windows XP platform and equipped with an X-ray microanalyzer (EDS INCA Energy TEM, Oxford Instruments, Bucks, UK) and an 11 Megapixel TEM Morada G2 camera (EMSIS GmbH, Muenster, Germany).

### 4.6. Statistical Analysis

The results of the microanalysis of Ca^2+^ cations in the fruit epidermis were analyzed statistically using univariate analysis of variance. The significance of differences was analyzed statistically using an integrated Statistica 13.1 statistical and analytical package. Univariate and multivariate analysis of variance (ANOVA) and Tukey comparison tests were performed. Statistical inference was carried out at the significance level of α = 0.05.

## 5. Conclusions

A beneficial effect of the applied calcium preparations was detected at the ultrastructural level in the epidermis and hypodermis cells in ‘Szampion’ apples. It was evidenced by the normally formed cell walls with a regular middle lamella outline, preserved continuity of the plasmalemma and tonoplast, and the presence of numerous amyloplasts, mitochondria, Golgi apparatus, and ER. This indicates stabilization of the cell ultrastructure, especially cell walls and cytoplasmic membranes, induced by Ca^2+^ ions. The highest level of Ca^2+^ in the selected fragments of the apical epidermis cells in the ‘Szampion’ apples was determined in the middle lamella, followed by the cell wall, plasmalemma, and cytoplasm. The microanalysis clearly showed higher Ca^2+^ concentrations in the examined fragments of epidermis cells in the plants from the calcium-supplemented treatments than in the control.

In light of the results of the present investigations, frequent application of calcium foliar feeding is recommended in order to ensure an optimal supply of this macronutrient in fruit, young leaves, and other intensively growing plant parts. The results obtained in the present study provide new information on plant biology, i.e., knowledge that can be useful in the field of plant nutrition. For the first time, microanalysis of calcium was carried out at the level of cell ultrastructure, and this element was mapped in the selected fragments of epidermal cells. This knowledge can be applied in cultivation work aimed at production of new high-quality apple varieties. In commercial production, it can help growers and managers to understand the fruit structure as a determinant of fruit capability of timely ripening, as well as firmness during storage and long-distance transport. The results presented in this paper indicate the need to use different forms of calcium in the foliar feeding of apple trees. The element increases the growth, yield, quality, and durability of fruit, and reduces their susceptibility to physiological disorders, e.g., bitter pit and lenticel blotch pit. These results may be important not only for researchers interested in plant biology and nutrition, but also for a wider group of readers.

There is a need for further research on the effect of Ca^2+^ cations originating from various calcium forms on improvement of cell firmness, enhancement of the integrity of cell walls and cytoplasmic membranes, and maintenance of the stability of membrane lipids and inhibition of peroxidation thereof, as they are important factors in fruit maturation, aging, and quality. It is necessary to continue investigations of various calcium forms in terms of the most favorable concentrations and stages of application to confirm their beneficial effects on the structure, commercial and storage quality, and the content of health-enhancing bioactive compounds in combination with their effectiveness in elimination or alleviation of physiological diseases. Finally, a possibility to reduce energy costs associated with prophylactic spraying applications and increase fruit production yields should be assessed.
